# The Alumni Association of the Buffalo Medical College

**Published:** 1883-03

**Authors:** 


### THE ALUMNI ASSOCIATION OF THE BUFFALO MEDICAL COLLEGE.

The ninth annual meeting of the Association was held at the Medical College building, Tuesday, Feb. 27th. President, Dr. Wm. Ring, in the Chair, and Dr. C. M. Daniels, Secretary.

The following alumni were present:

1847—James E. King, Buffalo; William Ring, Buffalo; Zera Hurd Blake, Dansville, N. Y.

1848—C. C. Wyckoff, Buffalo; G. H. Bennett, Lyman, N. Y.; Horace Hoyt, East Aurora, N. Y.

1851—Thomas D. Strong, Westfield, N. Y.

1852—John R. Cotes, Batavia, N. Y.

1859—W. W. Potter, Buffalo.

1860—C. H. Richmond, Livonia, N. Y.

1861—E. S. Carpenter, North Cohocton, N. Y.

1862—C. W. Coyler, Buffalo.

1865—H. Lapham, Clarence, N. Y.

1866—Conrad Diehl, Buffalo; M. B. Shaw, Eden, N. Y.

1867—E. C. W. O'Brien, Buffalo.

1870—C. B. Kipler, Corry, Pa.

1871—A. H Briggs, Buffalo; J. J. Walsh, Buffalo; D. W. Harrington, Buffalo.

1872—F. E. L. Brecht, Buffalo; J S. Halbert, Buffalo; Charles B. Knowlton, Buffalo; P. M. Wise, Willard, N. Y.

1873—W. H. Slacer, Buffalo; F. A. Burghardt, Buffalo; Joseph Fowler, Buffalo; C. R. Seeley, Attica, N. Y.

1874—Bernard Bartow, Buffalo; E. N. Brush, Utica

1875—William B. Clothier, Buffalo; D. T. Pierce, Argyle, N. Y.; F. A. Mandeville, Rochester.

1876—W. J. Packwood, Buffalo; A. A. Hubbell, Buffalo; B. A. Ellinworth, East Otto, N. Y.; Mrs. Mary B. Moody, Buffalo; W. D. Greene, Buffalo.

1877—Joseph Haberstro, Buffalo.

1878—Charles A. Ring, Buffalo Plains; Ernest Wende, Alden; C. F. Howard, Buffalo.

1879—Frederick Peterson, Buffalo; Eugene Storck, Buffalo.

1880—Mrs. M. J Slaight, Rochester; W. C. Barrett, Buffalo; Frank O. Vaughan, Buffalo; Edward Clark, Buffalo; C. H. Woodward, Bloods, N. Y.; C. M. Daniels, Buffalo; S. H. Warren, Buffalo; B. H Grove, Buffalo.

1881—J. J. Birmingham, Buffalo; William A. Hobday, Alton, Pa.; Mary E. Runner, Buffalo; C. C. Frederick, Buffalo.

1882—E. W. Wollaber, Cambria, N. Y; Willis G. Gregory, Buffalo; Eli H. Long, Buffalo; Frank H. Potter, Buffalo; J. W. Putnam, Buffalo.

The following officers were elected for the ensuing year:

President—Dr. E. N. Brush, Utica, N. Y.

First Vice-President—Dr. Horace Hoyt, East Aurora.

Second Vice-President—Dr. Conrad Diehl, Buffalo.

Third Vice-President—Dr. D. W. Harrington, Buffalo.

Fourth Vice-President—Dr. John H. Taylor, Holly, N. Y.

Fifth Vice-President—Dr. P. M. Wise, Ovid, N. Y.

Secretary—Dr. A. M. Barker, Buffalo.

Assistant Secretary—Dr. C. M. Daniels, Buffalo.

Trustee—Dr. C. C. Wyckoff.

Executive Committee—Dr. W. C. Barrett, Dr Bernard Bartow, Dr. E. C. W. O'Brien.

In the afternoon session Dr. P. M. Wise read an essay on "The Responsibilities of the Examining Physician on the Commitment of the Insane."

Dr. J. B. Andrews read a very able paper on "The Treatment of the Insane."

Dr. Frederick Peterson read an interesting paper on "The Alliptic Art."

